# Antibiotic Susceptibility and Technological Properties of *Leuconostoc citreum* for Selecting Starter Candidates

**DOI:** 10.3390/microorganisms12122636

**Published:** 2024-12-19

**Authors:** Sumin Lee, Sojeong Heo, Gawon Lee, Yura Moon, Minkyeong Kim, Mi-Sun Kwak, Do-Won Jeong

**Affiliations:** 1 Department of Food and Nutrition, Dongduk Women’s University, Seoul 02748, Republic of Korea; sumin4933@gmail.com (S.L.); hsjeong325v@gmail.com (S.H.); lgw9422@gmail.com (G.L.); moonyura20@gmail.com (Y.M.); alice09020104@gmail.com (M.K.); 2 Kookmin Bio Corporation, Seoul 02826, Republic of Korea; mskwak@kmbio.co.kr

**Keywords:** *Leuconostoc citreum*, antibiotic susceptibility, hemolysis, enzyme, starter

## Abstract

Antibiotic susceptibilities, hemolytic activities, and technological properties of 46 *Leuconostoc citreum* isolates from kimchi were evaluated to select starter candidates. All strains were susceptible to clindamycin and erythromycin, while some exhibited resistance to ampicillin, chloramphenicol, gentamicin, streptomycin, and tetracycline; all were resistant to kanamycin based on the EFSA breakpoint values for *Leuconostoc* species. PCR analysis did not detect resistance genes for these six antibiotics in any strain. None of the strains demonstrated clear α- or β-hemolytic activity. All strains thrived in a medium supplemented with 6% NaCl, displaying protease activity and acid in media containing 6% and 3% NaCl, respectively. Consequently, five strains, AK5T17, AK5T19, AK10M04, DMLC16, and YK10T20, were identified as starter candidates, with *L. citreum* strain DMLC16 emerging as the top choice due to its elevated protease and acid production capacities. These findings support the safe application of *L. citreum* strain DMLC16 as a starter candidate in fermented food production.

## 1. Introduction

The genus *Leuconostoc*, belonging to the order Lactobacillales, exhibits high morphological and physiological homogeneity [1]. Meanwhile, *Leuconostoc citreum* displays the significant characteristic of not metabolizing lactose [2] and demonstrates negative activities for α- and β-galactosidase and β-xylosidase. In contrast, *L. mesenteroides*, a representative species of the genus *Leuconostoc*, exhibits this activity [3]. *L. citreum* was originally isolated as a *L. mesenteroides* subsp. *amelibiosum*. Subsequently, DNA–DNA hybridization studies led to its reclassification as the new species, *L. amelibiosum*, from the *L. mesenteroides* subspecies [4]. It was later confirmed as another name for *L. citreum*, and ultimately classified under *L. citreum* [5]. Consequently, although *L. citreum* has been reclassified from *L. mesenteroides*, differences in enzyme activity suggest that *L. citreum*, when used as a starter culture, could produce fermentation products with characteristics distinct from those of *L. mesenteroides*.

The *Leuconostoc* genus is widely distributed in nature, including in fermented foods. Furthermore, *L. citreum* has been detected in various habitats, including fermented foods such as artisanal cheese [6], sourdough bread [7], and kimchi [8,9]. In industrial fermentation, *L. citreum* is used as a starter culture in the production of dairy products and fermented vegetables, primarily to enhance preservation through bacteriocin or acid production rather than through lactose fermentation [10,11,12]. *L. citreum* is a heterofermentative lactic acid bacterium that plays a crucial role in various food fermentations, enhancing flavor and texture [7,10,11,12].

Kimchi is a general term for a fermented vegetable dish that is widely consumed in Korea [13]. Among the various types of kimchi, Baechu-kimchi made from kimchi cabbage (*Brassica rapa*) is the most popular [14]. Kimchi is a fermented food produced through lactic acid fermentation, with microorganisms involved in this process primarily belonging to the genera *Leuconostoc*, *Lactobacillus*, and *Weissella* [15,16]. Among these, microorganisms of the genus *Leuconostoc*, particularly *L. mesenteroides*, are dominant during the early stages of fermentation. In the mid-fermentation stage, species from the genera *Lactobacillus* and *Weissella* dominate, while in the later stages of fermentation, acid-tolerant microorganisms such as the *Pediococcus* genus or yeasts become predominant [17].

In vegetable fermentation such as kimchi, heterofermentative lactic acid bacteria produce one molecule of lactic acid, one molecule of CO_2_, and one molecule of alcohol from one molecule of glucose. This process enhances the fresh and refreshing taste during the initial stages of fermentation. Due to this role, extensive efforts are made to promote heterofermentative bacteria in fermented vegetables like kimchi. One such strategy is employing starter cultures for fermentation [18]. The genus *Leuconostoc*, particularly *L. mesenteroides*, is frequently used as a starter culture in kimchi production. Commercial kimchi often utilizes this species. There have been recent attempts to incorporate other starter cultures alongside *L. mesenteroides*, such as *L. citreum*. In bread fermentation, *L. citreum* contributes to enhanced quality, nutritional profile, and sensory properties [7]. The use of *L. citreum* IH22 as a starter culture in kimchi was shown to delay the growth of homolactic-fermentative LAB, thereby lengthening shelf life [11]. A combination of *Lactococcus lactis* WiKim0098 and *L. citreum* WiKim0096 as a mixed starter culture has been shown to improve the shelf life and quality of kimchi [12]. Therefore, *L. citreum* displays strong potential as a fermentation starter culture.

It is advantageous for starter cultures in fermented foods to include a diverse range of candidates tailored to their specific purposes. For kimchi fermentation, efforts are being made to isolate and select starter cultures that can extend the optimal fermentation period, improve storage stability, and enhance health functionality, continuously [19,20]. In our previous study, 1219 LAB strains were isolated and identified from various kimchi types with added seafood to identify suitable starter cultures for kimchi fermentation [14,21]. The genus *Leuconostoc* was prevalent, with *L. citreum* (49 strains, 4.0%) being the second most frequently detected species after *L. mesenteroides* (482 strains, 39.5%). In this study, among previously isolated dominant species, *L. citreum* has been targeted for antibiotic resistance evaluation and enzyme activity assessment to isolate safe starter cultures with superior fermentation characteristics.

## 2. Materials and Methods

### 2.1. Bacterial Strains and Culture Conditions

A total of 46 *L. citreum* strains were utilized in this research after being definitively identified as *L. citreum* through reconfirmation of the entire 16S rRNA gene sequence from 49 *L. citreum* isolates (Table S1). These strains were obtained from commercial and traditional kimchi in previous studies analyzing the microbial community [14,21]. Additionally, the type strain *L. citreum* KACC 11860 was employed as a control. *Leuconostoc* species were cultured in de Man–Rogosa–Sharpe (MRS) broth (Becton, Dickinson and Co., Franklin Lakes, NJ, USA) at 30 °C for 24 h.

### 2.2. Antibiotic Susceptibility

Antibiotic susceptibility was assessed using the broth microdilution method [22] with eight antibiotics (Sigma-Aldrich, St. Louis, MO, USA): ampicillin, chloramphenicol, clindamycin, erythromycin, gentamicin, kanamycin, streptomycin, and tetracycline. Antibiotics were prepared in serial twofold working dilutions using deionized water, and antibiotic concentrations in each well of the 96-microwell plate ranged from 0.5 to 1024 mg/L. Bacterial strains were cultured twice in MRS broth and adjusted to a 0.5 McFarland turbidity standard (bioMérieux, Marcy L’Étoile, France). Subsequently, each suspension was diluted 1:100 in cation-adjusted Brain Heart Infusion (BHI) broth (Becton, Dickinson and Co.) to reach the appropriate inoculum concentration. The final inoculum density was 5 × 10^5^ colony-forming units/mL, and 200 µL were added to each well of the 96-microwell plate. The MICs of the antibiotics were identified as the lowest concentration preventing visible growth in the wells after incubation at 35 °C for 16 h. MIC results were verified by at least three independent tests. All experiments were performed at least three times on different days. Strains with MICs exceeding the breakpoint are considered resistant [23].

### 2.3. Identification of Antibiotic Resistance Gene

Genomic DNA from *L. citreum* strains was extracted using the DNeasy Tissue Kit (Qiagen, Hilden, Germany). Amplification of acquired antibiotic resistance genes was carried out using specific primer sets as listed in Table 1 with the T-3000 Thermocycler (Biometra, Göttingen, Germany). The PCR mixture comprised template DNA, 0.5 μM of each primer, 1.25 units of Inclone™ Taq polymerase (Inclone Biotech, Daejeon, Republic of Korea), 100 mM dNTPs, and 2.5 mM MgCl_2_. Samples were first preheated at 95 °C for 5 min and then subjected to 30 cycles of 1 min at 95 °C, 30 s at 57 °C, and 1 min at 72 °C. The PCR products were verified via migration on a 1.5% agarose gel stained with ethidium bromide. Additionally, for 4 out of the 9 genes, plasmids containing the genes were used as positive controls: pCL55 for *blaZ* gene*,* and *cat* gene [24]; pSSTET1 for *tetK* gene [25]; and pIMAY for *tetM* gene [26].

### 2.4. Hemolysis

The α- and β-hemolytic activities were assessed using Tryptic Soy Agar (TSA; Becton, Dickinson and Co.) supplemented with 5% (*v*/*v*) rabbit blood (MB Cell, Seoul, Republic of Korea) and 5% (*v*/*v*) sheep blood, respectively. α-Hemolytic activity was assessed after incubation at 30 °C for 24 h, while β-hemolytic activity was evaluated following a 24 h incubation at 30 °C with a subsequent cold shock at 4 °C. Hemolysis was identified by observing a clear lytic zone surrounding the colonies on each blood-containing TSA plate. Clinical strains *Staphylococcus aureus* USA300-p23 and RN4220 served as positive and negative controls, respectively. This experiment was repeated three times.

### 2.5. Determination of Salt Tolerance, Enzyme Activities, and Acid Production

Salt tolerance in *L. citreum* strains was evaluated by observing their growth on MRS media supplemented with 0–9% NaCl (*w*/*v*) as the final concentration. Growth was monitored over periods of 2, 4, 6, and 8 days.

Enzymatic activity for proteases and lipases was determined on TSA containing 0.5% (*w*/*v*) glucose and 2% (*w*/*v*) skim milk, and on tributyrin-agar containing 1% (*v*/*v*) tributyrin, respectively. The ability to produce acid was analyzed on TSA containing 0.5% (*w*/*v*) glucose and 0.7% (*w*/*v*) CaCO_3_. *L. citreum* cultures incubated at 30 °C for 24 h in MRS broth were applied to MRS agar to assess enzyme activity and acid productivity. The presence of enzyme activities and acid production were indicated by a clear halo around the disk containing the cultures. Additionally, the activity experiments were conducted independently with three repetitions, and the same results were obtained.

### 2.6. Strain Deposit

Strain *L. citreum* DMLC16 was deposited at the Korean Culture Center of Microorganisms under the accession number KFCC12011P.

## 3. Results

### 3.1. Antibiotic Resistance Profiles in Leuconostoc citreum Strains Were Analyzed

**Figure 1:**
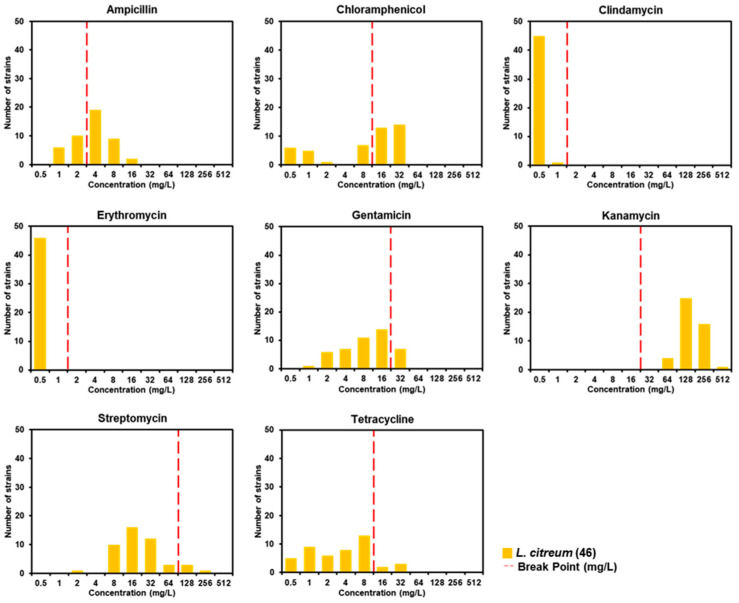
Minimum inhibitory concentration distributions for eight antibiotics and 46 *Leuconostoc citreum* strains isolated from kimchi, determined by the broth microdilution method. The vertical red dotted line represents the breakpoint for *Leuconostoc* spp., as established by EFSA [23].

To select antibiotic-sensitive strains, in accordance with the EFSA guidelines [23], resistance against eight antibiotics—ampicillin, chloramphenicol, clindamycin, erythromycin, gentamicin, kanamycin, streptomycin, and tetracycline—was determined for 46 *L. citreum* strains in this study. The antibiotic resistances, based on minimal inhibitory concentrations (MICs), were summarized (Figure 1). All 46 *L. citreum* strains were sensitive to clindamycin and erythromycin, while 30, 34, 7, 4, and 5 strains exhibited growth at concentrations exceeding the EFSA’s breakpoint values for ampicillin, chloramphenicol, gentamicin, streptomycin, and tetracycline, respectively (Figure 1). Notably, over half of the strains were resistant to ampicillin and chloramphenicol. Furthermore, all strains showed resistance to kanamycin, indicating that they all exceeded the EFSA breakpoint values for *Leuconostoc* species [23]. Consequently, five strains—AK5T17, AK5T19, AK10M04, DMLC16, and YK10T20—were identified that exhibited sensitivity to seven antibiotics, excluding kanamycin.

### 3.2. Absence of Acquired Antibiotic Resistance Genes in Leuconostoc citreum Strains

In the antibiotic susceptibility test, strain-specific resistance was observed for six out of the eight tested antibiotics (Figure 1). To determine whether these strains harbor acquired antibiotic resistance genes contributing to resistance against these six antibiotics, amplification was performed using primer sets designed to detect known acquired antibiotic resistance genes [27,28,29,30,31,32]. Nine genes associated with resistance to six antibiotics, known to be located on mobile elements such as plasmids and capable of being transferred to other strains, were targeted (Table 1). The presence of antibiotic resistance genes in 46 strains was examined using PCR amplification; however, none of the target genes were detected in any of the strains, including the five antibiotic-sensitive strains (Figure S1). These findings suggest that the 46 strains did not harbor the nine acquired antibiotic resistance genes that confer resistance to the six antibiotics.

### 3.3. Hemolysis of Leuconostoc citreum Strains

**Figure 2:**
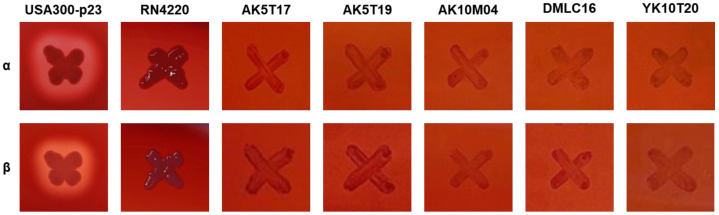
α-Hemolytic activity and β-hemolytic activity of five *Leuconostoc citreum* strains showing sensitivity to seven antibiotics. *Staphylococcus aureus* strains USA300-p23 and RN4220 were used as positive and negative controls, respectively.

The EFSA QPS guidelines for microorganisms used in food and feed, confirm that the absence of acquired antibiotic resistance is sufficient for *L. citreum*. However, typically, industrial practices also assess hemolytic activity in strains intended for food use. Therefore, we evaluated both α-hemolysis and β-hemolysis [33]. None of the strains, including the five antibiotic-sensitive strains, demonstrated hemolysis, unlike the positive control, *S. aureus* USA300-p23 (Figure 2).

### 3.4. Technological Property of Leuconostoc citreum Strains; Salt Tolerance, Protease, Lipase, and Amylase Activities, and Acid Production

According to the European Food & Feed Cultures Association, starter cultures are safe live bacteria, yeasts, or molds used in food production that contribute to storage and food quality, including sensory properties in the food [34]. Hence, it is essential that starter candidates not only be safe but also possess specific technological properties. To enhance the sensory attributes of fermented food, they must withstand the food environment and generate exoenzymes like amylase, proteases, and lipases. We assessed the salt tolerance, amylase activity, protease activity, lipase activity, and acid production capabilities of our *L. citreum* strains.

Salt tolerance is critical for applications in high-salt fermented foods such as *doenjang* (fermented soybean pastes) and *jeotgal* (fermented seafood). All the *L. citreum* strains grew well on MRS, with a 6% final concentration of NaCl (*w*/*w*) (Table 2), but failed to grow on media with 9% NaCl.

Amylase, protease, and lipase activities contribute to the sensory properties of fermented foods by producing taste and aroma compounds from starch, protein, and lipid constituents of food [35,36,37,38]. None of the strains exhibited amylase or lipase activity (Table 2). The protease activity of *L. citreum* was strain-specific, and fewer strains displayed protease activity as the NaCl concentration increased (Table 2). At 3% salt concentration, all but one strain showed protease activity; at 6% salt concentration, 31 strains (W, 16 strains; +, 13 strains; ++, 1 strain; +++, 1 strain) exhibited protease activity.

Acid production in fermented foods enhances both flavor and preservation. Similar to protease activity, acid production displayed strain-specific variations. When no additional salt was added, all strains showed some level of acid production. However, at a final concentration of 3% salt, 35 strains (W, 3 strains; +, 14 strains; ++, 18 strains) demonstrated acid production (Table 2).

## 4. Discussion

This study aimed to select starter culture candidate strains from *L. citreum* isolated from kimchi, focusing on strains that are antibiotic-sensitive, non-hemolytic, and exhibit desirable traits such as enzymatic activity and salt tolerance.

Food microorganisms used as starters, ingredients, and functional foods must not exhibit antibiotic resistance, particularly acquired antibiotic resistance [23,39]. The kimchi-derived *L. citreum* employed in this experiment is intended for use as a starter in fermented foods, requiring these strains to be antibiotic-sensitive. Consequently, we investigated the antibiotic resistance of *L. citreum* strains against eight antibiotics recommended by EFSA for *Leuconostoc* spp. [23], due to the absence of specific guidelines, such as those from the Clinical and Laboratory Standards Institute, the European Committee on Antimicrobial Susceptibility Testing, and the United States Committee on Antimicrobial Susceptibility Testing, for assessing antibiotic resistance in *L. citreum* [23]. Although five strains did not exhibit resistance to seven antibiotics, all *L. citreum* strains showed resistance to kanamycin, indicating that they all exceeded the EFSA breakpoint values for *Leuconostoc* species [23]. Findings similar to those concerning kanamycin resistance of *L. citreum* have been reported by Iosca et al. [40] and Flórez et al. [41], where all examined strains from both studies showed values surpassing EFSA’s kanamycin breakpoints for *Leuconostoc* spp. Both our current findings and prior research indicate that the EFSA kanamycin breakpoint for *Leuconostoc* spp. may vary for *L. citreum*. Consequently, it is essential to compile antibiotic resistance data specifically for *L. citreum* to determine more precise breakpoint values for this species.

Through MIC experiments, it was demonstrated that even if values exceed the cut-off, the strain can be used as a starter culture if the resistance is intrinsic [23,39]. Similarly, resistance due to genomic mutations is generally considered acceptable. However, if resistance is caused by the introduction of genes, it is classified as acquired and cannot be used. Gene introduction typically occurs through horizontal transfer involving mobile genetic elements like plasmids, making it a strain-specific characteristic [42,43,44]. In this study, certain strains exhibited values higher than the cut-off for six of the eight tested antibiotics. To investigate whether this resistance was due to the presence of acquired resistance genes, nine genes known to confer resistance to these six antibiotics were targeted and checked via PCR (Table 1). None of the strains were found to harbor these nine genes. However, it is important to note that the genes responsible for resistance are not limited to the nine tested. Further research is needed to identify resistance-contributing genes in *L. citreum* for these six antibiotics. In particular, identifying resistance genes for kanamycin is essential to determine whether the strains can be applied in the food industry.

**Figure 3:**
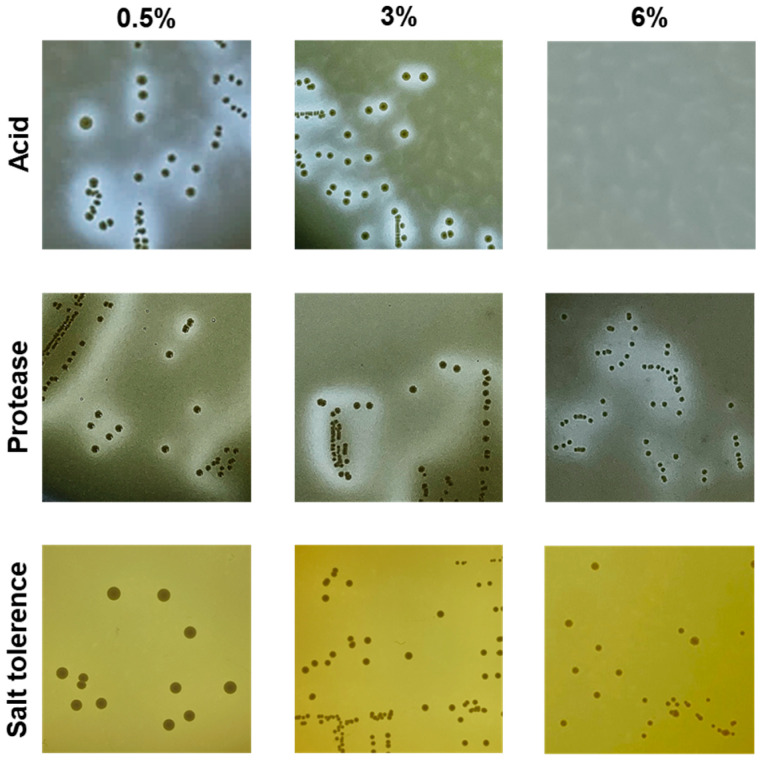
Salt tolerance and enzymatic activities of *Leuconostoc citreum* DMLC16. Acid and protease activities, along with salt tolerance, were assessed on TSA and MRS agar containing 0.5%, 3%, and 6% NaCl (*w*/*v*) as final concentrations.

Based on the results of the antibiotic sensitivity assessment and hemolysis, five strains assumed safe were selected (Table 3). However, since all selected strains exhibited resistance to kanamycin, it will be necessary to elucidate the mechanism of kanamycin resistance in future studies. All five strains grew on TSA medium containing 6% NaCl (*w*/*v*) and displayed protease activity and acid production in media containing 6% and 3% NaCl (*w*/*v*), respectively. Among these, DMLC16 was identified as the best candidate due to its lower MIC values for the seven antibiotics tested and its efficient technological properties (Figure 3 and Table 3).

## 5. Conclusions

The selected strain DMLC16, isolated from kimchi, has been confirmed to be safe, and its enzymatic activity is expected to be useful as a starter culture in fermented foods. In particular, the protease is expected to contribute to the sensory characteristics of protein-rich foods when used as a starter culture by producing amino acids through protein degradation. Therefore, further studies are recommended to evaluate the effects of DMLC16 in fermented foods as a starter culture. Additionally, if the health benefits of this strain are demonstrated, it may also have potential as a probiotic. Verifying these experimental results through genomic analysis would provide meaningful support for the findings. Furthermore, since there are no established resistance criteria for *L. citreum*, further research is needed to develop accurate standards for antibiotic resistance in this species.

## Figures and Tables

**Table 1:** Oligonucleotides for the identification of acquired antibiotic resistance genes.

Antibiotic	Target Gene	Oligonucleotide Sequence (5′-3′)		Size (bp)	Reference
		Forward	Reverse		
Ampicillin	*blaZ*	TACTTCAACACCTGCTGCTTTCG	ATTACACTCTTGGCGGTTTCAC	325	[27]
Chloramphenicol	*cat*	CCAGCAAACTACGTATAGCATTAC	GATGAAGCTGCAAGGCAACTGG	499	[28]
Gentamicin	*aac(6*′*)-aph(2*″*)*	CCAAGAGCAATAAGGGCATACC	ACCCTCAAAAACTGTTGTTGC	675	[29]
Kanamycin	*aphA-3*	GGGACCACCTATGATGTGGAACG	CAGGCTTGATCCCCAGTAAGTC	600	[30]
Streptomycin	*ant(6)*	ACTGGCTTAATCAATTTGGG	GCCTTTCCGCCACCTCACCG	597	[31]
	*str(A)*	CTTGGTGATAACGGCAATTC	CCAATCGCAGATAGAAGGC	500	[32]
	*str(B)*	ATCGTCAAGGGATTGAAACC	GGATCGTAGAACATATTGGC	500	[32]
Tetracycline	*tetK*	TTAGGTGAAGGGTTAGGTCC	GCAAACTCATTCCAGAAGCA	718	[28]
	*tetM*	ACAGAAAGCTTATTATATAAC	TGGCGTGTCTATGATGTTCAC	171	[28]

**Table 2:** Number of *Leuconostoc citreum* strains showing salt tolerance and enzyme activities under different NaCl concentrations.

Growth or Zone of Halo	NaCl (*w*/*v*) Tolerance		Protease Activity			Acid Production	
	3%	6%	0.5% ^a^	3%	6%	0.5% ^a^	3%
G	46	46					
—				1	15		11
W			1	8	16	1	3
+			11	25	13	3	14
++			29	7	1	42	18
+++			5	5	1		
Total	46	46	46	46	46	46	46

^a^ The NaCl concentration in TSA is 0.5% (*w*/*v*), and other values indicate the final concentration of NaCl in the medium. Abbreviations: G, growth. Diameter of growth-inhibition zone: —, 0.0 mm; W, <0.5 mm; +, 0.5–2.0 mm; ++, 2.0–4.0 mm; +++, >4.0 mm.

**Table 3:** Technological properties of five selected *Leuconostoc citreum* strains.

Strain	NaCl (*w*/*v*) Tolerance		Protease ^a^			Acid ^a^	
	3%	6%	0.5% ^b^	3%	6%	0.5%	3%
AK5T17	G	G	+++	+++	+	++	W
AK5T19	G	G	+++	+++	W	++	W
AK10M04	G	G	+++	+++	++	++	+
DMLC16	G	G	+++	+++	+++	++	++
YK10T20	G	G	+++	+++	W	++	+

^a^ Diameter of clear zone: W, <0.5 mm; +, 0.5–2.0 mm; ++, 2.0–4.0 mm; +++, >4.0 mm; ^b^ The NaCl concentration in TSA is 0.5% (*w*/*v*), with other values reflecting the final NaCl concentration in the medium. Abbreviations: G, growth.

## Data Availability

The original contributions presented in this study are included in the article/Supplementary Materials. Further inquiries can be directed to the corresponding author.

## Supplementary Materials

The following supporting information can be downloaded at: https://www.mdpi.com/article/10.3390/microorganisms12122636/s1, Table S1: List of 46 Strains of *Leuconostoc citreum* Isolated from Various Regions and Types of Kimchi; Figure S1: Identification of antibiotic resistance gene using PCR amplification.
