# Supplementary material for: Antibiotic Susceptibility and Technological Properties of Leuconostoc citreum for Selecting Starter Candidates
Source: Microorganisms. 2024 Dec 19;12(12):2636. doi: 10.3390/microorganisms12122636 (PMC11679923; doi:10.3390/microorganisms12122636)
Supplement: Supplementary file 1 [file microorganisms-12-02636-s001.zip › microorganisms-3353360-supplementary.pdf]

Table S1. List of 46 Strains of *Leuconostoc citreum* Isolated from Various Regions and Types of Kimchi.

Origin	Number of strains	Region	Reference
Baechu-kimchi	1	Donghae	[1]
Myeongtae-baechu-kimchi	7	Donghae	[1]
Gul-baechu-kimchi	3	Gumi	[1]
Jeonbok-baechu-kimchi	21	Namyangju	[1]
Baechu-kimchi	8	Sangju	[2]
Jogi-baechu-kimchi	6	Sangju	[2]
Total	46		

Abbreviations: C: Control

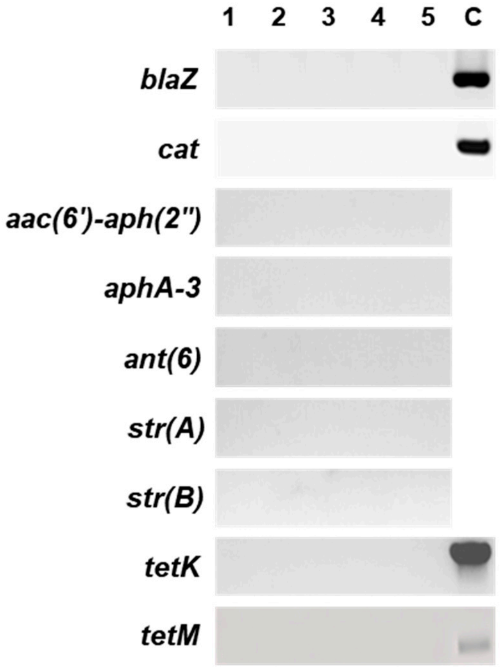

Figure S1. Identification of antibiotic resistance gene using PCR amplification .

Lanes 1, strain AK5T17; 2, strain AK5T19; 3, strain AK10M04; 4, strain DMLC16; 5, strain YK10T20; C, positive control. pCL55 for *blaZ,* and *cat* [3]; pSSTET1 for *tetK* [4]; pIMAY for *tetM* [5] were used as positive controls, respectively.

References

1. Park, J.; Heo, S.; Lee, G.; Hong, S. W.; Jeong, D.-W. Bacterial community of kimchi added with seafood based on culture-dependent investigations. *Heliyon*. **2024**, *10*, e34153, https://doi.org/10.1016/j.heliyon.2024.e34153.
2. Park, J.; Heo, S.; Na, H.-E.; Lee G, Kim, T, Sung, M.-H. Jeong, D.-W. Culture-dependent and -independent investigations of the effect of addition of jogi on the bacterial community of kimchi. *Food Biosci*. **2023**, *54*, 102832, https://doi.org/10.1016/j.fbio.2023.102832.
3. Lee, C.Y.; Buranen, S.L.; Zhi-Hai, Y. Construction of single-copy integration vectors for *Staphylococcus aureus*. *Gene*. **1991**, *103*, 101–105, https://doi.org/10.1016/0378-1119(91)90399-V
4. Lee, J.-H.; Heo, S.; Jeong, M.; Jeong, D.-W. Transfer of a mobile *Staphylococcus saprophyticus* plasmid isolated from fermented seafood that confers tetracycline resistance. *PLoS ONE*. **2019**, *14*, e0213289, https://doi.org/10.1371/journal.pone.0213289
5. Monk, I.R.; Shah, I.M.; Xu, M.; Tan, M.W.; Foster, T.J. Transforming the untransformable: Application of direct transformation to manipulate genetically *Staphylococcus aureus* and *Staphylococcus epidermidis*. *mBio*. **2012**, *3*, e00277-11, https://doi.org/10.1128/mbio.00277-11.
